# Early-pregnancy trunk phase angle derived from bioelectrical impedance analysis for the prediction of gestational diabetes mellitus

**DOI:** 10.3389/fendo.2026.1870018

**Published:** 2026-08-14

**Authors:** Qun Ji, Boxia Fu, Lan Gao, Haiwei Liu, Yikai Liu, Huibiao Quan, Fei Wang

**Affiliations:** Department of Endocrinology, Hainan General Hospital, Hainan Affiliated Hospital of Hainan Medical University, Haikou, Hainan, China

**Keywords:** early prediction, gestational diabetes mellitus, gestational weight gain, HOMA-IR, triglycerides, trunk phase angle

## Abstract

**Objective:**

To evaluate first-trimester predictors of gestational diabetes mellitus (GDM) and develop an early risk model.

**Methods:**

Women at 8–12 weeks of gestation who attended routine antenatal care at Hainan General Hospital between October 2023 and December 2024 were enrolled and followed until 24–28 gestational weeks for a 75-g oral glucose tolerance test (OGTT). Participants were classified into a GDM group or a normal glucose tolerance (NGT) group according to standard diagnostic criteria. General clinical characteristics, fasting biochemical parameters, and body-composition variables were collected in early pregnancy; Body composition was measured at 13 weeks using a multi-frequency bioelectrical impedance analyzer (InBody 770). Logistic regression, ROC, bootstrap validation, calibration plots, and decision curve analysis were used.

**Results:**

Of 125 women (68 GDM, 57 NGT), GDM had higher age, pre-pregnancy body mass index (p-BMI), assisted conception, family hyperglycemia, first-trimester weight gain, glucose, insulin, HOMA-IR, TyG, lipids, neutrophils, uric acid, and most body-composition indices (all P<0.05). After adjustment, 25 variables (P<0.01) were grouped into six domains. HOMA-IR, triglycerides (TG), first-trimester gestational weight gain (GWG-F), and 50-kHz trunk phase angle (trunk PhA) were selected as core predictors. The BIC-based model identified HOMA-IR (OR = 3.287), TG (OR = 2.277), GWG-F (OR = 1.150), trunk phase angle (OR = 2.615), and age (OR = 1.180) as independent risk factors. The model showed good discrimination (AUC = 0.921), calibration (Hosmer–Lemeshow P = 0.287), and net benefit.

**Conclusion:**

HOMA-IR, TG, GWG-F, trunk PhA, and age are independent early GDM predictors. The combined model (AUC = 0.921) can help identify high-risk women early for timely intervention.

## Introduction

Gestational diabetes mellitus (GDM) has increased rapidly worldwide in recent years (1, 2). In China, the prevalence of GDM has continued to rise and it has become one of the most common metabolic disorders during pregnancy (3). Previous investigations into the etiology of GDM have primarily focused on traditional risk factors, including pre-pregnancy overweight or obesity, advanced maternal age, family history of diabetes, and gestational weight gain (GWG) (4). Although screening and management systems for GDM in China have become increasingly comprehensive, the disease burden remains substantial. Therefore, a better understanding of GDM pathogenesis and the implementation of early-pregnancy risk assessment are essential for preventing GDM and related adverse maternal and neonatal outcomes.

Early pregnancy is a critical metabolic window during which alterations in glucose and lipid metabolism may influence the later development of GDM. Biochemical markers such as blood glucose, lipid indices, and insulin levels are important for assessing maternal metabolic status. Studies have shown that age, ethnicity, pre-pregnancy obesity, and family history of diabetes are established risk factors for GDM (5, 6), and that elevated fasting glucose, total cholesterol, and triglycerides are also associated with increased GDM risk (7, 8). However, even among women with similar pre-pregnancy characteristics, early-pregnancy biochemical markers and body-composition profiles may differ and may contribute differently to subsequent GDM risk.

At present, evidence regarding the influence of early-pregnancy body-composition characteristics, such as body fat percentage and muscle mass, on GDM remains limited. Most existing anthropometric studies have focused on traditional indicators, including body mass index (BMI), waist-to-hip ratio, body fat percentage (9, 10), and GWG (11). Notably, one detailed anthropometric study showed that each one-unit increase in first-trimester neck circumference was associated with a 1.29-fold increase in the risk of GDM (12). Other studies have also reported significant associations between GDM risk and neck circumference, waist circumference, hip circumference, arm circumference, waist-to-hip ratio, visceral fat depth, subcutaneous fat depth, and short stature. These findings suggest that an early-pregnancy prediction model integrating traditional risk factors with multidimensional body-composition parameters may improve the identification of women at high risk for GDM later in pregnancy. Machine learning approaches have shown promise in early GDM prediction (e.g., Hassan et al., AUC up to 98.71%), supporting the development of integrated clinical and body-composition models (13).

Bioelectrical impedance analysis (BIA) is a convenient, rapid, and noninvasive method for assessing body composition, including muscle mass, visceral fat, and intracellular and extracellular water (14). Because it is noninvasive and radiation-free (15), BIA is particularly suitable for large-scale studies and clinical assessment in women of reproductive age (16). From BIA, a raw parameter termed phase angle (PhA) can be derived, calculated as the arctangent of the ratio of reactance (Xc) to resistance (R) at a given frequency, i.e., PhA = arctan(Xc/R) × 180/π. PhA reflects the phase shift between voltage and current as they pass through biological tissues and is considered a marker of cell membrane integrity, cellular health, and the relative distribution of intra- and extracellular water. Lower PhA values have been associated with impaired cell function, increased inflammation, and poorer nutritional status in various metabolic conditions, whereas higher PhA is generally interpreted as better cell integrity and larger fat-free mass. Indeed, PhA has been linked to multiple metabolic disorders (17), with previous studies reporting lower PhA in patients with type 2 diabetes and obesity compared with healthy controls. Previous studies have shown that PhA is lower in patients with type 2 diabetes than in healthy controls (18–20), and a reduced PhA has also been reported in obesity (21). PhA has additionally been proposed as a noninvasive marker of nutritional status during pregnancy (22). However, data on the characteristics of PhA in women with GDM and on its relationship with GDM development remain scarce. Therefore, this study aimed to investigate the independent roles of biochemical markers and BIA-derived body-composition features, particularly PhA, in the early development of GDM, and to clarify their associations with GDM risk in order to provide a basis for early prediction and intervention.

## Materials and methods

### Study population

This prospective cohort study included women in early pregnancy (8–12 gestational weeks) who attended routine antenatal care at Hainan General Hospital between October 2023 and December 2024. All participants were followed until 24–28 gestational weeks and underwent a 75-g OGTT. According to the OGTT results, participants were classified into the normal glucose tolerance (NGT) group or the GDM group.

Inclusion criteria were as follows: (1) diagnosis of GDM according to the International Association of Diabetes and Pregnancy Study Groups (IADPSG) criteria (13), defined as at least one of the following at 24–28 gestational weeks: fasting plasma glucose ≥ 5.1 mmol/L, 1-h plasma glucose ≥ 10.0 mmol/L, or 2-h plasma glucose ≥ 8.5 mmol/L after the 75-g OGTT; (2) age ≥ 18 years; (3) singleton pregnancy; (4) voluntary participation with written informed consent and willingness to comply with regular antenatal follow-up and OGTT testing; and (5) ability to understand the study procedures and complete data collection, including questionnaire assessment, physical examination, and BIA measurement.

Exclusion criteria were: (1) pre-existing diabetes or overt diabetes diagnosed during pregnancy; (2) multiple pregnancy; (3) thyroid dysfunction or autoimmune disease; (4) infectious disease; (5) polycystic ovary syndrome; (6) use of medications affecting glucose metabolism within the previous 3 months; (7) other pregnancy complications or severe heart, liver, or kidney failure; (8) psychiatric disease or cognitive impairment; (9) malignant tumor; and (10) incomplete pregnancy data or an anticipated high risk of loss to follow-up.

This study was approved by the Medical Ethics Committee of Hainan General Hospital (No. [2023]253 and No. Med-Eth-Re[2025]).

## Data collection

All variables were collected at the first assessment in early pregnancy.

General clinical information included age, self-reported pre-pregnancy weight, height, pre-pregnancy BMI (pre-BMI), education level, conception mode, medical history, and family history.

Biochemical parameters were measured according to standard laboratory procedures and included fasting plasma glucose, white blood cell count, neutrophil count, monocyte count, red blood cell count, hemoglobin, platelet count, albumin, prealbumin, aspartate aminotransferase, alanine aminotransferase, total cholesterol, high-density lipoprotein cholesterol, low-density lipoprotein cholesterol, lymphocyte count, eosinophil count, basophil count, urea, uric acid, creatinine, and estimated glomerular filtration rate. Participants fasted for 8–10 h before blood sampling.

Body composition was measured at a standardized time point of 13 weeks of gestation using a multi-frequency bioelectrical impedance analyzer (InBody 770; InBody Co., Ltd., Korea). This timing standardizes early-pregnancy assessment, minimizes variability from gestational fluid shifts, and follows protocols used in previous studies. Before the BIA test, participants were instructed to avoid food and water intake, empty the bladder, remove shoes and socks, wear clothing of known weight, and remove all metallic objects. Participants stood barefoot on the device platform, held the hand electrodes with the arms extended downward in a neutral position, placed all five fingers on the hand electrodes, and positioned the heels and forefeet on the foot electrodes. The device directly recorded the following parameters: total body water, protein, minerals, body fat mass, muscle mass, visceral fat area, waist-to-hip ratio, bone mineral content, skeletal muscle mass, skeletal muscle index, fat mass index, fat-free mass index, trunk fat percentage, trunk fat-to-fat-free mass ratio, limb fat-free mass percentage, 50-kHz whole-body phase angle(whole-body PhA), 50-kHz trunk phase angle(trunk PhA), 50-kHz right arm phase angle(right arm PhA), 50-kHz left arm phase angle(left arm PhA), 50-kHz right-leg phase angle(right leg PhA), 50-kHz left-leg phase angle(left leg PhA), and the InBody score.

First-trimester gestational weight gain (GWG-F) was calculated as body weight at 13 gestational weeks minus self-reported pre-pregnancy weight. BMI was calculated as weight (kg)/height (m)^^2^.

## Statistical analysis

All statistical analyses were performed using R version 4.5.0. Normality of continuous variables was assessed with the Shapiro–Wilk test. Normally distributed variables are presented as mean ± standard deviation (SD) and were compared between groups using the independent-samples t-test. Non-normally distributed variables are presented as median (interquartile range) and were compared using the Mann–Whitney U test. Categorical variables are presented as n (%) and were compared using the chi-square test or Fisher’s exact test, as appropriate.

Group comparisons between women with GDM and NGT were performed using the independent-samples t-test or Mann–Whitney U test for continuous variables and the chi-square test or Fisher’s exact test for categorical variables, as appropriate. Because a large number of variables were analyzed, multiple comparisons were performed. To control the false discovery rate (FDR) and reduce the risk of false-positive findings, the Benjamini–Hochberg procedure was applied to adjust the P values across all comparisons in **Tables 1**–**3**. Statistical significance was defined as an FDR-adjusted P value < 0.05.

**Table 1:** Baseline characteristics of the study participants.

Variables	Total (n=125)	NGT (n=57)	GDM (n=68)	P	FDR-adjusted P
Age, years	32(28,35)	29 (27,33)	33(30,35.25)	<0.001^a^	<0.001
Pre-BMI kg/m²	21.78 (19.63,23.47)	21.38 (19.05,22.66)	22.31 (20.50,24.54)	0.005^a^	0.009
Education				0.583^b^	0.625
Junior high school and below	20 (16%)	7 (12.3%)	13 (19.1%)		
Special Secondary School/senior high school	19 (15.2%)	9 (15.8%)	10 (14.7%)		
Junior college/undergraduate	86 (68.8%)	41 (71.9%)	45 (66.2%)		
Pregnancy mode				0.002^b^	0.003
Spontaneous pregnancy	97 (77.6%)	52 (91.2%)	45 (66.2%)		
Assisted pregnancy	28 (22.4%)	5 (8.8%)	23 (33.8%)		
Hypertension				0.986^b^	0.986
Yes	12 (9.6%)	6 (10.5%)	6 (8.8%)		
No	113 (90.4%)	51 (89.5%)	62 (91.2%)		
Dyslipidemia				0.289	0.328
Yes	8 (6.4%)	2 (3.5%)	6 (8.8%)		
No	117 (93.6%)	55 (96.5%)	62 (91.2%)		
Elevated blood glucose				0.007^b^	0.010
No family history	82 (65.6%)	42 (73.7%)	40 (58.8%)		
Second-degree relatives	22 (17.6%)	12 (21.1%)	10 (14.7%)		
First-degree relatives	21 (16.8%)	3 (5.3%)	18 (26.5%)		

Data are presented as median (Q1, Q3) or n (%), as appropriate. Q1 and Q3 denote the first and third quartiles, respectively.

GDM, gestational diabetes mellitus; NGT, normal glucose tolerance; Pre-pregnancy BMI, kg/m², pre-pregnancy body mass index. Hypertension was defined as presence (Yes) or absence (No) of hypertension. Dyslipidemia was defined as presence (Yes) or absence (No) of dyslipidemia. Family history of elevated blood glucose was categorized as no family history, second-degree relatives only, or first-degree relatives. First-degree relatives included parents, full siblings, and children, whereas second-degree relatives included grandparents, grandchildren, aunts and uncles, nieces and nephews, and half-siblings. Participants with an affected first-degree relative were classified into the first-degree relative category, regardless of the presence of affected second-degree relatives.

P values were adjusted for multiple comparisons using the Benjamini–Hochberg procedure across all tests in **Tables 1**–**3** to control the false discovery rate. For categorical variables, adjustment was performed at the variable level. Statistical significance was defined as an FDR-adjusted P value < 0.05. ^a^Mann–Whitney U test; ^b^Pearson chi-square test; ^c^Fisher exact test.

**Table 2:** Routine hematologic and biochemical indices of the study participants.

Variables	Total (n = 125)	NGT (n = 57)	GDM (n = 68)	P	FDR-adjusted P
GWG-F, kg	3.65 ± 4.93	1.42 ± 3.64	5.51 ± 5.11	<0.001^a^	<0.001
T1-BMI,kg/m2	23.40 ± 3.85	21.46 ± 3.15	25.02 ± 3.65	<0.001^b^	<0.001
FPG, mmol/L	4.57 (4.30,5.11)	4.36 (4.15,4.61)	4.84 (4.43,5.32)	<0.001^a^	<0.001
Insulin, pmol/L	74.79 (50.14,108.50)	57.30 (42.75,75.02)	96.00 (69.84,125.60)	<0.001^a^	<0.001
HOMA-IR	2.33 (1.43,3.35)	1.56 (1.16,2.31)	3.04 (2.29,4.10)	<0.001^a^	<0.001
TyG index	8.86 (8.32,9.23)	8.43 (7.99,8.84)	9.13 (8.81,9.42)	<0.001^a^	<0.001
WBC, ×10^9^/L	9.24 (7.75,10.84)	9.08 (7.54,10.48)	9.87 (8.12,10.83)	0.086^a^	0.108
NEUT, ×10^9^/L	6.87 ± 1.79	6.51 ± 1.79	7.17 ± 1.75	0.041^b^	0.053
MONO, ×10^9^/L	0.51 (0.43,0.62)	0.49 (0.41,0.59)	0.53 (0.44,0.62)	0.099^a^	0.123
LYM, ×10^9^/L	1.92 (1.59,2.20)	1.97 (1.65,2.24)	1.88 (1.59,2.14)	0.382^a^	0.425
EOS, ×10^9^/L	0.09 (0.07,0.18)	0.08 (0.05,0.16)	0.10 (0.07,0.18)	0.266^a^	0.308
BASO, ×10^9^/L	0.02 (0.02,0.03)	0.02 (0.02,0.04)	0.02 (0.01,0.03)	0.216^a^	0.253
RBC, × 1012/L	4.22 (3.85,4.58)	4.31 (3.96,4.66)	4.10 (3.73,4.47)	0.104^a^	0.126
Hb, g/L	120.58 ± 11.99	123.00 ± 12.02	118.54 ± 11.66	0.038^b^	0.050
PLT, ×10^9^/L	258.00 (228.00,298.00)	261.00 (238.00,301.00)	251.00 (218.75,297.25)	0.324^a^	0.364
TC, mmol/L	5.33 (4.57,6.09)	4.79 (4.22,5.52)	5.70 (4.98,6.49)	<0.001^a^	<0.001
TG, mmol/L	1.95 (1.12,2.61)	1.37 (0.87,1.95)	2.27 (1.87,2.96)	<0.001^a^	<0.001
HDL-C, mmol/L	2.05 (1.81,2.35)	1.91 (1.67,2.18)	2.08 (1.90,2.44)	0.026^a^	0.036
LDL-C, mmol/L	2.77 (2.20,3.38)	2.54 (2.18,3.02)	3.03 (2.55,3.57)	0.001^a^	0.002
NLR	3.56 (2.84,4.55)	3.27 (2.66,4.18)	3.86 (3.19,4.57)	0.024^a^	0.035
PLR	142.05 (115.38,175.22)	138.82 (118.95,167.58)	143.54 (112.19,181.46)	0.596^a^	0.635
MHR	0.25 (0.21,0.31)	0.25 (0.21,0.31)	0.24 (0.21,0.31)	0.794^a^	0.821
Urea, mmol/L	2.55 (2.20,3.03)	2.50 (2.12,2.94)	2.66 (2.35,3.16)	0.110^a^	0.133
Uric acid, μmol/L	230.00 (196.00,273.00)	218.00 (187.00,255.00)	251.50 (209.75,290.75)	0.008^a^	0.012
Creatinine, μmol/L	40.00 (36.00,46.00)	40.00 (37.00,45.00)	40.00 (34.75,46.00)	0.479^a^	0.529
eGFR, mL/min/1.73m²	172.80 (147.93,197.71)	172.88 (150.02,191.27)	168.45 (147.58,202.24)	0.876^a^	0.891

Data are presented as mean ± standard deviation (SD), or median (Q1, Q3), as appropriate. Q1 and Q3 denote the first and third quartiles, respectively. GWG-F, first-trimester gestational weight gain; T1-BMI, First-trimester BMI; FPG, fasting plasma glucose; HOMA-IR, homeostasis model assessment of insulin resistance; TyG, triglyceride-glucose index; WBC, white blood cell count; NEUT, neutrophil count; MONO, monocyte count; LYM, lymphocyte count; EOS, eosinophil count; BASO, basophil count; RBC, red blood cell count; Hb, hemoglobin; PLT, platelet count; TC, total cholesterol; TG, triglycerides; HDL-C, high-density lipoprotein cholesterol; LDL-C, low-density lipoprotein cholesterol; NLR, neutrophil-to-lymphocyte ratio; PLR, platelet-to-lymphocyte ratio; MHR, monocyte-to-high-density lipoprotein cholesterol ratio; eGFR, glomerular filtration rate.

P values were adjusted for multiple comparisons using the Benjamini–Hochberg procedure across all tests in **Tables 1**–**3** to control the false discovery rate. For categorical variables, adjustment was performed at the variable level. Statistical significance was defined as an FDR-adjusted P value < 0.05. ^a^Mann–Whitney U test; ^b^ Independent samples t-test.

**Table 3:** Body-composition parameters of the study participants.

Variables	Total (n = 125)	NGT (n = 57)	GDM (n = 68)	P	FDR-adjusted P
TBW, L	27.99 ± 3.61	26.69 ± 2.91	29.08 ± 3.78	<0.001^a^	<0.001
ICW, L	17.16 ± 2.26	16.33 ± 1.82	17.85 ± 2.38	<0.001^a^	<0.001
ECW, L	10.83 ± 1.35	10.36 ± 1.11	11.23 ± 1.42	<0.001^b^	<0.001
Protein, kg	7.24 ± 0.99	7.06 ± 0.79	7.72 ± 1.04	<0.001^a^	<0.001
Minerals, kg	2.81 ± 0.37	2.69 ± 0.31	2.92 ± 0.38	<0.001^b^	<0.001
BFM, kg	20.75 ± 6.35	18.19 ± 5.21	22.90 ± 6.45	<0.001^b^	<0.001
LBM, kg	35.87 ± 4.65	34.18 ± 3.75	37.28 ± 4.88	<0.001^a^	<0.001
FFM, kg	38.22 ± 4.94	36.43 ± 3.99	39.72 ± 5.18	<0.001^a^	<0.001
SMM, kg	20.38 ± 2.95	19.30 ± 2.37	21.28 ± 3.09	<0.001^a^	<0.001
BMI, kg/m²	23.40 ± 3.85	21.47 ± 3.15	25.02 ± 3.65	<0.001^b^	<0.001
PBF, %	34.49 ± 5.41	32.69 ± 5.50	35.99 ± 4.88	<0.001^b^	<0.001
Right arm fat-free mass, kg	1.80 ± 0.40	1.65 ± 0.32	1.93 ± 0.41	<0.001^b^	<0.001
Right arm fat-free mass percentage, %	91.16 ± 12.15	86.38 ± 9.65	95.17 ± 12.63	<0.001^a^	<0.001
Left arm fat-free mass, kg	1.75 ± 0.39	1.59 ± 0.31	1.89 ± 0.40	<0.001^b^	<0.001
Left arm fat-free mass percentage, %	88.55 ± 11.8	83.43 ± 9.17	92.84 ± 12.10	<0.001^a^	<0.001
Trunk fat-free mass, kg	16.95 ± 2.38	16.08 ± 1.90	17.68 ± 2.50	<0.001^a^	<0.001
Trunk fat-free mass percentage, %	95.34 ± 6.10	93.53 ± 5.00	96.85 ± 6.54	0.002^a^	0.003
Right leg fat-free mass, kg	5.75 ± 0.83	5.55 ± 0.71	5.92 ± 0.89	0.014^b^	0.022
Right leg fat-free mass percentage, %	91.70 (88.40,96.60)	92.20 (88.40,95.60)	91.50 (89.02,97.03)	0.783^a^	0.818
Left leg fat-free mass, kg	5.73 ± 0.83	5.54 ± 0.73	5.89 ± 0.88	0.018^b^	0.026
Left leg fat-free mass percentage, %	91.70 (88.30,96.20)	91.60 (88.30,96.10)	91.70 (88.20,96.70)	0.791^a^	0.819
TBW of right arm, L	1.40 ± 0.31	1.28 ± 0.25	1.51 ± 0.32	<0.001^b^	<0.001
TBW of left arm, L	1.37 ± 0.30	1.24 ± 0.24	1.47 ± 0.31	<0.001^a^	<0.001
TBW of trunk, L	13.23 ± 1.85	12.56 ± 1.49	13.80 ± 1.94	<0.001^a^	<0.001
TBW of right leg, L	4.49 ± 0.65	4.34 ± 0.56	4.62 ± 0.69	0.015^b^	0.023
TBW of left leg, L	4.48 ± 0.65	4.33 ± 0.57	4.60 ± 0.69	0.020^b^	0.028
ICW of right arm, L	0.87 ± 0.19	0.80 ± 0.15	0.93 ± 0.20	<0.001^b^	<0.001
ICW of left arm, L	0.85 ± 0.19	0.77 ± 0.15	0.91 ± 0.19	<0.001^b^	<0.001
ICW of trunk, L	8.10 ± 1.16	7.68 ± 0.93	8.46 ± 1.22	<0.001^a^	<0.001
ICW of right leg, L	2.75 ± 0.40	2.65 ± 0.34	2.83 ± 0.43	0.011^b^	0.016
ICW of left leg, L	2.74 ± 0.40	2.64 ± 0.35	2.82 ± 0.43	0.014^b^	0.021
ECW of right arm, L	0.53 ± 0.12	0.49 ± 0.09	0.57 ± 0.12	<0.001^b^	<0.001
ECW of left arm, L	0.52 ± 0.11	0.47 ± 0.09	0.56 ± 0.12	<0.001^a^	<0.001
ECW of trunk, L	5.13 ± 0.70	4.88 ± 0.56	5.34 ± 0.73	<0.001^a^	<0.001
ECW of right leg, L	1.74 ± 0.25	1.69 ± 0.22	1.78 ± 0.27	0.029^b^	0.04
ECW of left leg, L	1.74 ± 0.25	1.69 ± 0.23	1.78 ± 0.26	0.036^b^	0.048
Extracellular water ratio, %	0.39 (0.38,0.39)	0.39 (0.38,0.39)	0.39 (0.38,0.39)	0.071^a^	0.092
Right arm extracellular water ratio, %	0.38 ± 0.01	0.38 ± 0.01	0.38 ± 0.01	0.054^a^	0.070
Left arm extracellular water ratio, %	0.38 (0.38,0.38)	0.38 (0.38,0.38)	0.38 (0.38,0.38)	0.024^a^	0.034
Trunk extracellular water ratio, %	0.39 (0.38,0.39)	0.39 (0.38,0.39)	0.39 (0.38,0.39)	0.186^a^	0.222
Right leg extracellular water ratio, %	0.39 (0.38,0.39)	0.39 (0.38,0.39)	0.39 (0.38,0.39)	0.103^a^	0.126
Left leg extracellular water ratio, %	0.39 ± 0.01	0.39 ± 0.01	0.39 ± 0.01	0.078^b^	0.099
Right arm fat mass, kg	1.4 (1.2,1.8)	1.30 (1.00,1.50)	1.60 (1.30,1.90)	<0.001^a^	<0.001
Right arm fat percentage, %	158.20 (130.3,198.3)	138.70 (118.40,163.20)	180.20 (147.43,222.78)	<0.001^a^	<0.001
Left arm fat mass, kg	1.4 (1.2,1.8)	1.30 (1.10,1.50)	1.65 (1.30,2.00)	<0.001^a^	<0.001
Left arm fat percentage, %	161.60 (134.80,205.30)	145.80 (122.80,166.30)	183.25 (150.30,227.55)	<0.001^a^	<0.001
Trunk fat mass, kg	10.20 ± 3.36	8.83 ± 2.84	11.35 ± 3.34	<0.001^b^	<0.001
Trunk fat percentage, %	205.20 ± 66.10	175.66 ± 56.86	229.96 ± 63.41	<0.001^b^	<0.001
Right leg fat mass, kg	3.22 ± 0.89	2.87 ± 0.75	3.51 ± 0.90	<0.001^b^	<0.001
Right leg fat percentage, %	142.35 ± 38.33	125.59 ± 33.03	156.39 ± 37.00	<0.001^b^	<0.001
Left leg fat mass, kg	3.21 ± 0.89	2.86 ± 0.75	3.50 ± 0.90	<0.001^b^	<0.001
Left leg fat percentage, %	141.83 ± 38.26	125.19 ± 33.06	155.78 ± 36.92	<0.001^b^	<0.001
InBody score	69.00 (66.00,72.00)	69.00 (66.00,71.00)	69.00 (66.00,72.00)	0.764^a^	0.804
WHR	0.88 ± 0.05	0.87 ± 0.04	0.89 ± 0.05	0.032^b^	0.043
VFA, cm²	103.26 ± 35.85	90.39 ± 30.64	114.04 ± 36.54	<0.001^b^	<0.001
Degree of obesity, %	111.54 ± 18.42	102.19 ± 14.96	119.37 ± 17.45	<0.001^b^	<0.001
BCM, kg	24.57 ± 3.25	23.38 ± 2.61	25.57 ± 3.42	<0.001^a^	<0.001
Arm circumference, cm	28.58 ± 3.15	27.05 ± 2.65	29.86 ± 2.98	<0.001^b^	<0.001
Arm muscle circumference, cm	24.20 (22.40,25.30)	23.00 (21.50,24.30)	24.70 (23.50,26.02)	<0.001^a^	<0.001
BMC, kg	2.35 ± 0.30	2.25 ± 0.26	2.44 ± 0.31	<0.001^b^	<0.001
Total body water/fat-free mass ratio	73.20 (73.10,73.30)	73.30 (73.10,73.40)	73.20 (73.10,73.30)	0.211^a^	0.249
FFMI, kg/m²	15.16 ± 1.69	14.31 ± 1.40	15.88 ± 1.58	<0.001^b^	<0.001
FMI, kg/m²	8.24 ± 2.46	7.15 ± 2.06	9.14 ± 2.40	<0.001^b^	<0.001
Right arm PhA, °	4.34 ± 0.52	4.15 ± 0.44	4.50 ± 0.53	<0.001^b^	<0.001
Left arm PhA, °	4.11 ± 0.51	3.91 ± 0.45	4.28 ± 0.51	<0.001^b^	<0.001
Trunk PhA, °	5.60 (5.30,6.00)	5.40 (5.20,5.80)	5.80 (5.30,6.32)	0.003^a^	0.004
Right leg PhA, °	5.41 ± 0.77	5.11 ± 0.74	5.66 ± 0.72	<0.001^b^	<0.001
Left leg PhA, °	4.78 ± 0.67	4.57 ± 0.64	4.96 ± 0.64	0.001^b^	0.002
Whole-body PhA, °	4.62 ± 0.54	4.42 ± 0.49	4.79 ± 0.53	<0.001^b^	<0.001
Neck circumference, cm	32.3 ± 2.75	31.26 ± 2.23	33.17 ± 2.85	<0.001^b^	<0.001
Chest circumference, cm	87.19 ± 7.07	84.00 ± 5.75	89.87 ± 7.00	<0.001^b^	<0.001
Waist circumference, cm	81.82 ± 9.18	78.27 ± 7.35	84.79 ± 9.56	<0.001a	<0.001
Hip circumference, cm	93.01 ± 6.18	90.11 ± 5.06	95.44 ± 6.01	<0.001^b^	<0.001
Right arm circumference, cm	28.82 ± 3.19	27.29 ± 2.68	30.11 ± 3.03	<0.001^b^	<0.001
Left arm circumference, cm	28.58 ± 3.15	27.05 ± 2.65	29.86 ± 2.98	<0.001^b^	<0.001
Right thigh circumference, cm	49.85 ± 4.11	47.93 ± 3.46	51.46 ± 3.94	<0.001^b^	<0.001
Left thigh circumference, cm	49.65 ± 4.07	47.77 ± 3.44	51.23 ± 3.89	<0.001^b^	<0.001
SMI, kg/m²	5.96 ± 0.75	5.62 ± 0.61	6.23 ± 0.74	<0.001^b^	<0.001

Data are presented as mean ± standard deviation (SD), or median (Q1, Q3), as appropriate. Q1 and Q3 denote the first and third quartiles, respectively.

TBW, total body water; ICW, intracellular water; ECW, extracellular water; FFM, fat-free mass; SMM, skeletal muscle mass; BFM, body fat mass; LBM, Lean body mass; PBF, percent body fat; FMI, fat mass index; FFMI, fat-free mass index; WHR, waist-to-hip ratio; VFA, visceral fat area; BCM, body cell mass; BMC, bone mineral content; SMI, skeletal muscle mass index; PhA, phase angle. All PhA measurements were obtained at a frequency of 50 kHz and are expressed in degrees (°).

Raw two-sided P values are shown. P values from all variable-level comparisons across **Tables 1**–**3** were adjusted using the Benjamini–Hochberg procedure to control the false discovery rate. For categorical variables, each variable was treated as a single hypothesis. An FDR-adjusted P value < 0.05 was considered statistically significant. ^a^Mann–Whitney U test; ^b^independent samples t-test.

Minimally adjusted binary logistic regression was used to evaluate hematologic, biochemical, and body-composition variables, with age and pre-pregnancy BMI included as covariates. Variables with P < 0.01 were grouped according to biological plausibility, and one clinically representative variable from each category was retained to construct the multivariable logistic regression model. This strategy was used to control for multiple comparisons and mitigate overfitting. An Enter model and a bidirectional stepwise model based on the Bayesian information criterion (BIC) were compared, and the optimal model was selected.

Model discrimination was assessed using ROC curves and the C-index (AUC) with 95% confidence intervals. Internal validation was performed using bootstrap resampling (1,000 repetitions), and calibration was evaluated using bias-corrected calibration curves, the Hosmer–Lemeshow goodness-of-fit test (g = 10), the Brier score, and the calibration slope. Decision curve analysis (DCA) and a nomogram were used to assess clinical utility. All tests were two-sided, and P < 0.05 was considered statistically significant. Due to the single-center, exploratory design and limited sample size, only internal validation via bootstrap was performed. External validation should be addressed in future studies.

## Results

**Figure 1:**
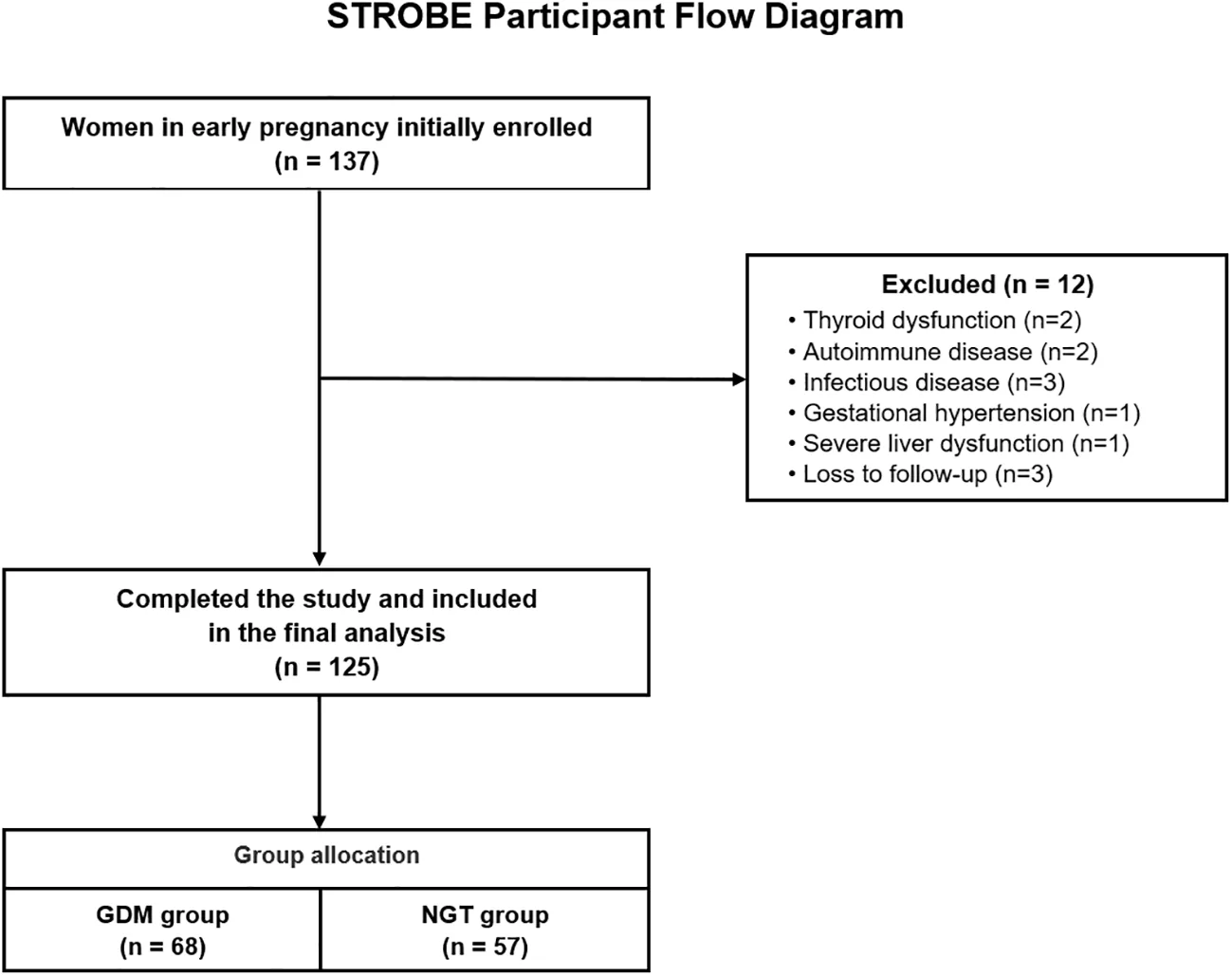
STROBE participant flow diagram.

GDM, Gestational Diabetes Mellitus; NGT, Normal Glucose Tolerance. This diagram follows the STROBE guidelines for reporting participant flow in observational studies. A total of 137 women in early pregnancy were initially enrolled. Twelve participants were excluded for the reasons listed above. The remaining 125 women completed the study and were included in the final analysis (68 in the GDM group and 57 in the NGT group).

A total of 125 women in early pregnancy completed the study and were included in the final analysis, including 68 in the GDM group and 57 in the NGT group (**Figure 1**). Among the 137 women initially enrolled, 12 were excluded because of thyroid dysfunction (n=2), autoimmune disease (n=2), infectious disease (n=3), gestational hypertension (n=1), severe liver dysfunction (n=1), or loss to follow-up (n=3). Comparisons of baseline characteristics, biochemical indices, and body-composition parameters between the two groups are presented below.

### Baseline characteristics

Mann–Whitney U, chi-square, and Fisher’s exact tests showed significant between-group differences in age, pre-pregnancy BMI, conception mode, and family history of hyperglycemia (all P < 0.05). Compared with the NGT group, women in the GDM group were older, had a higher pre-BMI, were more likely to have conceived through assisted reproduction, and were more likely to report a first-degree family history of hyperglycemia. No significant between-group differences were observed in education level, hypertension, or dyslipidemia (all P > 0.05) (**Table 1**).

### Routine hematologic and biochemical indices

Compared with the NGT group, the GDM group showed significantly greater GWG-F, higher first-trimester BMI (T1- BMI), fasting plasma glucose (FPG), insulin, HOMA-IR, TyG, neutrophil count, total cholesterol(TC), TG, HDL-C, LDL-C, neutrophil-to-lymphocyte ratio(NLR), and uric acid, as well as lower hemoglobin levels (all P< 0.05). No significant between-group differences were observed in white blood cell count, monocyte count, lymphocyte count, eosinophil count, basophil count, red blood cell count, platelet count, platelet-to-lymphocyte ratio (PLR), monocyte-to-HDL ratio (MHR), urea, creatinine, or estimated glomerular filtration rate(eGFR) (all P>0.05) (**Table 2**).

### Body composition parameters

No significant between-group differences were found for the fat-free mass (FFM) percentage of right leg, fat-free mass percentage(FFM%) of left leg, Extracellular Water (ECW)/Total Body Water (TBW), ECW/TBW of the right arm, ECW/TBW of the trunk, ECW/TBW of both legs, the InBody score, or TBW/FFM (all P > 0.05). In contrast, several body-composition measures, including multiple 50-kHz PhA parameters, were significantly higher in the GDM group than in the NGT group (P < 0.05) (**Table 3**).

### Minimally adjusted logistic regression analysis of risk factors for GDM

In the univariable analyses (**Supplementary Table S1**), a number of variables showed significant associations with GDM (P < 0.05). To control for potential confounding by age and pre‑pregnancy BMI, each of these variables was then entered separately into minimally adjusted binary logistic regression models. With GDM status as the binary dependent variable (GDM = 1, NGT = 0), variables that differed significantly in the univariable analyses were entered separately as independent variables into minimally adjusted binary logistic regression models, with age and pre-BMI included as covariates. Twenty-five variables remained significant at P < 0.01, spanning domains related to glucose–lipid metabolism, overall adiposity, body-fat distribution, anthropometry, and phase angle. Full results are presented in **Table 4**. Definitions of all candidate variables are provided in **Table 5**.

**Table 4:** Minimally adjusted logistic regression analysis of risk factors for GDM.

Variable	B	OR	95% CI, Lower	95% CI, Upper	P
GWG-F, kg	0.204	1.226	1.111	1.369	<0.001
Insulin, pmol/L	0.031	1.032	1.018	1.049	<0.001
HOMA-IR	1.183	3.265	2.059	5.616	<0.001
FPG, mmol/L	2.628	13.845	4.675	49.936	<0.001
TG, mmol/L	0.963	2.621	1.626	4.529	<0.001
TC, mmol/L	0.681	1.975	1.395	2.965	<0.001
TyG index	1.825	6.201	2.755	15.595	<0.001
First-trimester BMI, kg/m²	0.533	1.704	1.329	2.256	<0.001
Degree of obesity, %	0.113	1.120	1.063	1.187	<0.001
FFMI, kg/m²	0.738	2.092	1.413	3.240	<0.001
Arm circumference, cm	0.548	1.729	1.289	2.414	0.001
Right leg PHA, °	1.043	2.837	1.597	5.431	0.001
Right arm circumference, cm	0.529	1.697	1.268	2.362	0.001
Left arm circumference, cm	0.548	1.729	1.289	2.414	0.001
Arm muscle circumference, cm	0.570	1.769	1.253	2.605	0.002
Hip circumference, cm	0.217	1.242	1.092	1.432	0.002
Right thigh circumference, cm	0.265	1.303	1.107	1.557	0.002
Left thigh circumference, cm	0.267	1.306	1.106	1.566	0.002
Trunk fat percentage, %	0.022	1.022	1.008	1.037	0.003
FMI, kg/m²	0.542	1.720	1.214	2.524	0.003
Trunk PHA, °	0.856	2.353	1.350	4.420	0.004
Right leg fat percentage, %	0.028	1.028	1.009	1.050	0.005
Chest circumference, cm	0.176	1.193	1.059	1.361	0.005
Left leg fat percentage, %	0.028	1.028	1.009	1.049	0.006
LDL-C, mmol/L	0.762	2.143	1.257	3.828	0.007
Left arm fat-free mass percentage, %	0.064	1.066	1.018	1.122	0.010
Right arm fat percentage, %	0.018	1.018	1.005	1.033	0.010
Left arm PHA, °	1.237	3.445	1.397	9.508	0.011
Left arm fat percentage, %	0.018	1.018	1.004	1.033	0.013
SMI, kg/m²	1.098	2.998	1.297	7.472	0.013
Right arm PHA, °	1.013	2.755	1.17	7.099	0.026
Whole-body PHA, °	1.009	2.743	1.158	7.075	0.027
NLR	0.366	1.443	1.069	2.040	0.030
ICW of left arm, L	3.821	45.631	1.640	1754.678	0.031
Weight, kg	0.076	1.079	1.008	1.162	0.035
Right arm fat-free mass percentage, %	0.048	1.050	1.005	1.100	0.035
Left arm fat-free mass, kg	1.772	5.881	1.188	33.878	0.037
TBW of left arm, L	2.287	9.846	1.255	94.022	0.037
BFM, kg	0.136	1.146	1.012	1.313	0.039
Trunk fat mass, kg	0.267	1.306	1.023	1.705	0.039
Uric acid, μmol/L	0.007	1.007	1.001	1.014	0.041
Right arm fat mass, kg	1.427	4.166	1.101	18.403	0.048
Right leg fat mass, kg	0.801	2.227	1.037	5.126	0.048
ECW of left arm, L	5.614	274.191	1.254	98127.1	0.050
Hb, g/L	-0.033	0.968	0.934	1.000	0.055
Trunk fat-free mass percentage, %	0.071	1.073	1.000	1.158	0.056
Left leg fat mass, kg	0.759	2.136	1.001	4.876	0.059
NEUT, ×10^9^/L	0.213	1.238	0.996	1.559	0.060
ICW of right arm, L	2.949	19.084	0.820	574.365	0.076
Right arm fat-free mass, kg	1.414	4.113	0.888	21.585	0.081
TBW of right arm, L	1.799	6.045	0.849	50.377	0.082
Left arm extracellular water ratio, %	-107.774	0.000	0.000	83962.530	0.083
HDL-C, mmol/L	0.880	2.410	0.946	6.873	0.087
ECW of right arm, L	4.564	95.932	0.536	25792.06	0.095
Left leg PHA, °	0.630	1.878	0.898	4.103	0.101
Left arm fat mass, kg	1.120	3.065	0.859	12.704	0.104
Minerals, kg	1.095	2.989	0.815	11.943	0.107
SMM, kg	0.151	1.163	0.971	1.412	0.112
ICW, L	0.196	1.216	0.962	1.565	0.113
BCM, kg	0.136	1.146	0.973	1.366	0.114
BMC, kg	1.301	3.673	0.759	19.559	0.114
Protein, kg	0.446	1.562	0.912	2.790	0.115
Waist circumference, cm	0.069	1.072	0.985	1.173	0.119
SMM, kg	0.092	1.096	0.979	1.237	0.121
FFM, kg	0.086	1.090	0.980	1.220	0.121
TBW, L	0.116	1.123	0.972	1.312	0.127
ECW, L	0.275	1.316	0.906	1.957	0.159
ICW of trunk, L	0.369	1.446	0.880	2.469	0.159
Trunk fat-free mass, kg	0.176	1.192	0.938	1.543	0.163
TBW of trunk, L	0.221	1.248	0.917	1.737	0.172
Neck circumference, cm	0.178	1.195	0.928	1.561	0.175
ECW of trunk, L	0.526	1.693	0.767	3.932	0.204
VFA, cm²	0.013	1.013	0.994	1.033	0.204
PBF, %	0.063	1.065	0.954	1.195	0.273
WHR	-3.755	0.023	0.000	1140.571	0.492
ICW of right leg, L	0.260	1.297	0.416	4.184	0.656
Right leg fat-free mass, kg	0.103	1.108	0.645	1.934	0.711
ICW of left leg, L	0.209	1.232	0.398	3.921	0.718
TBW of right leg, L	0.125	1.133	0.566	2.309	0.725
Left leg fat-free mass, kg	0.084	1.088	0.635	1.883	0.760
TBW of left leg, L	0.097	1.102	0.554	2.219	0.783
ECW of right leg, L	0.180	1.197	0.207	7.142	0.841
ECW of left leg, L	0.124	1.132	0.201	6.521	0.888

GDM was coded as 1 and NGT as 0. Each candidate variable was entered separately into a binary logistic regression model, minimally adjusted for maternal age and pre-pregnancy BMI. Variables were selected from those showing statistical significance in univariate analysis. B, unstandardized regression coefficient; OR, odds ratio; CI, confidence interval. The lower and upper limits refer to the 95% CI for the OR. Statistical significance was defined as P < 0.01.

**Table 5:** Definitions of variables included in the minimally adjusted logistic regression analyses for gestational diabetes mellitus.

Variable (Abbreviation), Unit	Definition
Age, years	Maternal age at the time of study enrollment
Pre-pregnancy BMI, kg/m²	Body mass index calculated using pre-pregnancy weight and height
First-trimester BMI, kg/m²	Body mass index calculated using First-trimester pregnancy weight and height
GWG-F, kg	Gestational weight gain during the first trimester
FPG, mmol/L	Fasting plasma glucose concentration measured in early pregnancy
Insulin, pmol/L	Fasting serum insulin concentration
HOMA-IR	Homeostasis model assessment of insulin resistance
TyG index	Triglyceride-glucose index
WBC, ×10^9^/L	White blood cell count
NEUT, ×10^9^/L	Neutrophil count
MONO, ×10^9^/L	Monocyte count
LYM, ×10^9^/L	Lymphocyte count
NLR	Neutrophil-to-lymphocyte ratio
PLR	Platelet-to-lymphocyte ratio
MHR	Monocyte-to-HDL cholesterol ratio
Urea, mg/dL	Serum urea concentration, a marker of renal function.
Uric acid, μmol/L	Serum uric acid concentration
Creatinine, μmol/L	Serum creatinine concentration
GFR, mL/min/1.73m²	Estimated glomerular filtration rate
TBW, L	Total body water
ICW, L	Intracellular water
ECW, L	Extracellular water
Protein, kg	Total body protein content
Minerals, kg	Total body mineral content
BFM, kg	Body fat mass
SMM, kg	Skeletal muscle mass
FFM, kg	Fat-free mass
PBF, %	Percent body fat
FMI, kg/m²	Fat mass index
FFMI, kg/m²	Fat-free mass index
BCM, kg	Body cell mass
BMC, kg	Bone mineral content
VFA, cm²	Visceral fat area
WHR	Waist-to-hip ratio
SMI, kg/m²	Skeletal muscle mass index
ICW of right arm, L	Intracellular water content of the right arm
ICW of left arm, L	Intracellular water content of the left arm
ICW of trunk, L	Intracellular water content of the trunk
ICW of right leg, L	Intracellular water content of the right leg
ICW of left leg, L	Intracellular water content of the left leg
ECW of right arm, L	Extracellular water content of the right arm
ECW of left arm, L	Extracellular water content of the left arm
ECW of trunk, L	Extracellular water content of the trunk
ECW of right leg, L	Extracellular water content of the right leg
ECW of left leg, L	Extracellular water content of the left leg
TBW of right arm, L	Total body water content of the right arm
TBW of left arm, L	Total body water content of the left arm
TBW of trunk, L	Total body water content of the trunk
TBW of right leg, L	Total body water content of the right leg
TBW of left leg, L	Total body water content of the left leg
Right arm fat-free mass, kg	Fat-free mass of the right arm
Left arm fat-free mass, kg	Fat-free mass of the left arm
Trunk fat-free mass, kg	Fat-free mass of the trunk
Right leg fat-free mass, kg	Fat-free mass of the right leg
Left leg fat-free mass, kg	Fat-free mass of the left leg
Right arm fat mass, kg	Fat mass of the right arm
Left arm fat mass, kg	Fat mass of the left arm
Trunk fat mass, kg	Fat mass of the trunk
Right leg fat mass, kg	Fat mass of the right leg
Left leg fat mass, kg	Fat mass of the left leg
Right arm fat percentage, %	Fat percentage of the right arm
Left arm fat percentage, %	Fat percentage of the left arm
Trunk fat percentage, %	Fat percentage of the trunk
Right leg fat percentage, %	Fat percentage of the right leg
Left leg fat percentage, %	Fat percentage of the left leg

### Multivariable logistic regression model for predicting GDM

Variables with P<0.01 in the minimally adjusted analyses were further classified according to clinical relevance into the following domains: (1) insulin resistance/glucose–lipid metabolism; (2) overall adiposity/body composition; (3) body-fat distribution; (4) anthropometric measurements; (5) phase angle; and (6) first-trimester gestational weight gain. To avoid overfitting, variable selection was guided by the events-per-variable (EPV) principle (EPV≥10), and only the most clinically representative indicator from each domain was retained.

Because composite indices such as HOMA-IR and TyG already integrate information from fasting glucose, insulin, and lipid parameters, they were prioritized over individual component variables to reduce collinearity. In addition, because pre-pregnancy BMI was included as a covariate, body-composition and anthropometric variables with strong collinearity with BMI were excluded. After this screening process, four core predictors were retained: HOMA-IR, TG, GWG-F, and trunk PhA.

These core predictors were entered into multivariable unconditional logistic regression models with age and pre-pregnancy BMI as covariates. Two model-building strategies were used: an Enter model including all candidate variables and a bidirectional stepwise model based on the BIC (implemented with the step AIC function in the MASS package, using k = ln[n]). Model fit was assessed with the Hosmer–Lemeshow test, discrimination with the AUC, and model comparison with the likelihood ratio test (LRT).

In the Enter model, six candidate variables were included: HOMA-IR, TG, GWG-F, trunk PhA, age, and pre-pregnancy BMI. Based on BIC-guided bidirectional stepwise selection, pre-pregnancy BMI was removed and a simplified model containing five variables was obtained (**Table 6**).

**Table 6:** Enter-based multivariable logistic regression analysis for predicting GDM.

Variable	b	Sb	Wald χ²	P	β	OR	95%CI Lower	95%CI Upper
Regression intercept	-14.238	3.949	13.001	<0.001	/	/	/	/
HOMA-IR	1.295	0.314	17.040	<0.001	1.966	3.651	1.974	6.753
TG	0.845	0.303	7.769	0.005	0.941	2.327	1.285	4.216
GWG-F	0.133	0.061	4.789	0.029	0.654	1.142	1.014	1.286
Trunk PHA	1.032	0.375	7.582	0.006	0.796	2.807	1.346	5.853
Age	0.177	0.072	6.053	0.014	0.775	1.194	1.037	1.376
Pre-pregnancy BMI	-0.100	0.109	0.845	0.358	-0.326	0.905	0.730	1.120

GDM was coded as 1 and NGT as 0. Multivariable logistic regression analysis was performed with gestational diabetes mellitus (GDM) as the dependent variable (GDM = 1, NGT = 0). All independent variables were entered into the model simultaneously using the Enter method. B, unstandardized regression coefficient; SE, standard error of the regression coefficient; Wald χ², Wald chi-square statistic; β, standardized regression coefficient; OR, odds ratio; 95% CI Lower and Upper, lower and upper bounds of the 95% confidence interval for the odds ratio. All P values were two-sided. p value < 0.05 is considered statistically significant.

The LRT showed no significant difference in fit between the full Enter model and the simplified stepwise model (difference in deviance = 0.855, df = 1, P = 0.355). Because the simplified model achieved a higher EPV (13.6 vs. 11.3) without compromising explanatory performance, the BIC-based stepwise model was selected as the final model.

In the final BIC-based multivariable logistic regression model, HOMA-IR, TG, GWG-F, trunk PhA, and age were independent risk factors for GDM (OR = 3.287, 95% CI: 1.961–6.191, P < 0.001; OR = 2.277, 95% CI: 1.333–4.336, P = 0.006; OR = 1.150, 95% CI: 1.027–1.304, P = 0.021; OR = 2.615, 95% CI: 1.315–5.689, P = 0.009; and OR = 1.180, 95% CI: 1.033–1.367, P = 0.019, respectively) (**Table 7**).

**Table 7:** BIC-based stepwise multivariable logistic regression analysis for predicting GDM.

Variable	b	Sb	Wald χ²	P	β	OR	95%CI Lower	95%CI Upper
Regression intercept	-15.378	3.885	15.665	<0.001	/	/	/	/
HOMA-IR	1.190	0.291	16.770	<0.001	1.806	3.287	1.961	6.191
TG	0.823	0.297	7.695	0.006	0.916	2.277	1.333	4.336
GWG-F	0.139	0.060	5.341	0.021	0.688	1.150	1.027	1.304
Trunk PHA	0.961	0.365	6.920	0.009	0.741	2.615	1.315	5.689
Age	0.165	0.071	5.463	0.019	0.722	1.180	1.033	1.367

GDM was coded as 1 and NGT as 0. Candidate predictors were evaluated using stepwise binary logistic regression with BIC as the model-selection criterion. BIC, Bayesian information criterion; B, unstandardized regression coefficient; SE, standard error of the regression coefficient; Wald χ², Wald chi-square statistic; β, standardized regression coefficient; OR, odds ratio; 95% CI Lower and Upper, lower and upper bounds of the 95% confidence interval for the odds ratio. All P values were two-sided. P value < 0.05 is considered statistically significant.

### Predictive performance of the model

**Figure 2:**
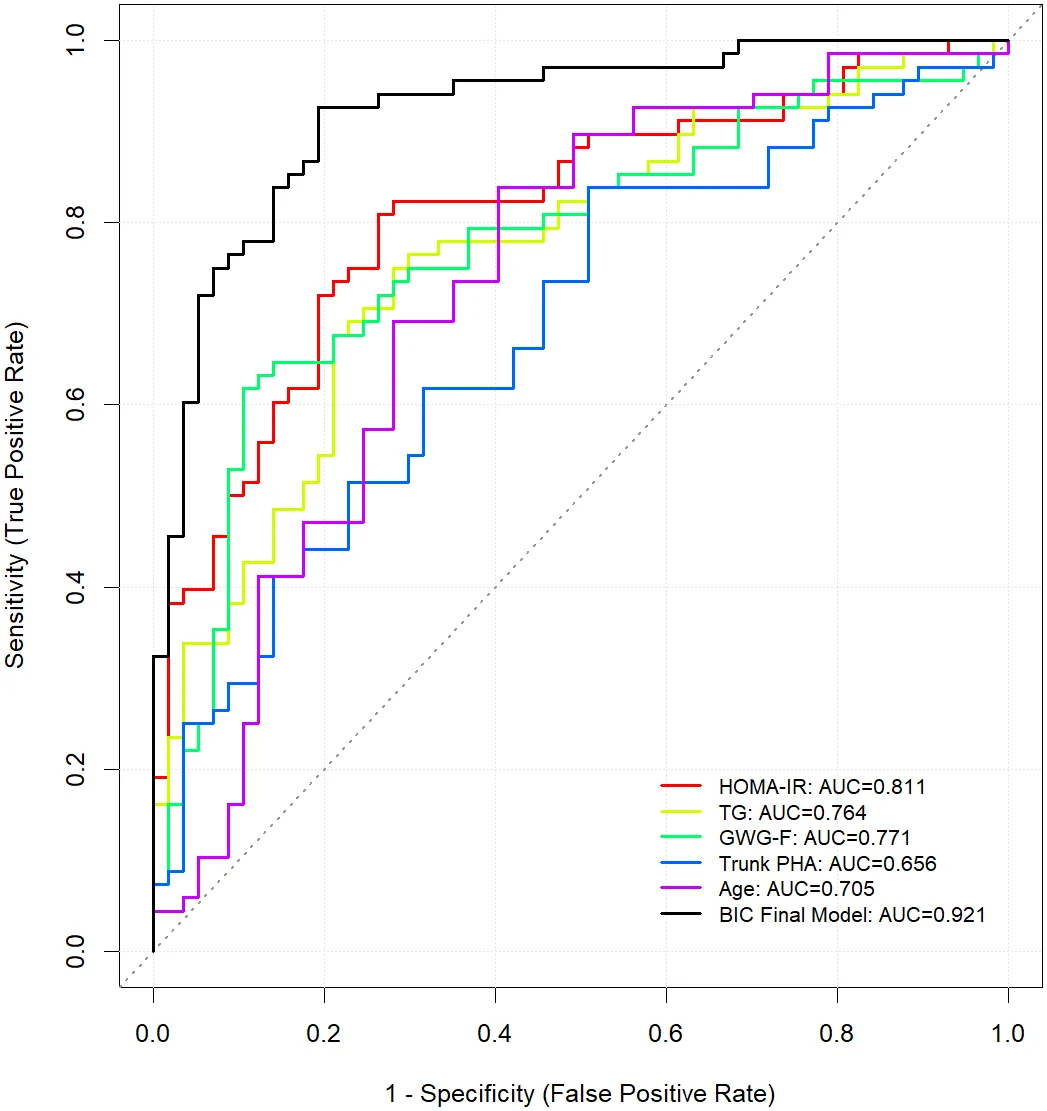
ROC curves of individual indicators and the combined model for predicting GDM.

ROC analysis was performed with GDM status as the binary outcome variable and age, HOMA-IR, TG, GWG-F, and trunk PhA as predictors. The combined model showed the best predictive performance, with an AUC of 0.921 (95% CI: 0.874–0.968; P < 0.001), sensitivity of 0.926, specificity of 0.807, and an optimal cutoff value of 0.440. Among individual indicators, HOMA-IR had the strongest predictive ability (AUC = 0.811, 95% CI: 0.736–0.887; P < 0.001), followed by GWG-F (AUC = 0.771, 95% CI: 0.686–0.855; P < 0.001) and TG (AUC = 0.764, 95% CI: 0.681–0.848; P < 0.001). Trunk PhA and age showed more modest performance, with AUCs of 0.656 (95% CI: 0.561–0.752; P = 0.001) and 0.705 (95% CI: 0.610–0.799; P < 0.001), respectively (**Table 8**; **Figure 2**).

**Table 8:** Predictive performance of individual variables and the combined model for GDM.

Variable	AUC	SE	95% CI Lower	95%CI Upper	P	Sensitivity	Specificity	Youden’s index	Cut-off
Model	0.921	0.024	0.874	0.968	<0.001	0.926	0.807	0.733	0.440
HOMA-IR	0.811	0.039	0.736	0.887	<0.001	0.809	0.737	0.546	0.473
TG	0.764	0.043	0.681	0.848	<0.001	0.750	0.719	0.469	0.537
GWG-F	0.771	0.043	0.686	0.855	<0.001	0.618	0.895	0.512	0.620
Trunk PHA	0.656	0.049	0.561	0.752	0.001	0.412	0.825	0.236	0.603
Age	0.705	0.048	0.610	0.799	<0.001	0.838	0.509	0.347	0.457

Model: The final predictive model for gestational diabetes mellitus (GDM) was developed using BIC-based stepwise multivariate logistic regression, with maternal age and pre-pregnancy BMI forced into the model. AUC, area under the ROC curve; SE, standard error; CI, confidence interval; Youden’s index = sensitivity + specificity − 1. The optimal cut-off value for each variable was determined by maximizing Youden’s index. P value indicates whether the AUC is significantly different from 0.5. P value < 0.01 is considered statistically significant.

### Internal validation and calibration

The final model showed good fit, with a Hosmer–Lemeshow P value of 0.287, indicating good agreement between predicted and observed outcomes. The final AIC was 101.7, suggesting that the model achieved adequate fit without unnecessary complexity. The model also demonstrated excellent discrimination (AUC = 0.921, 95% CI: 0.874–0.968), and the EPV of 11.3 supported model stability.

**Figure 3:**
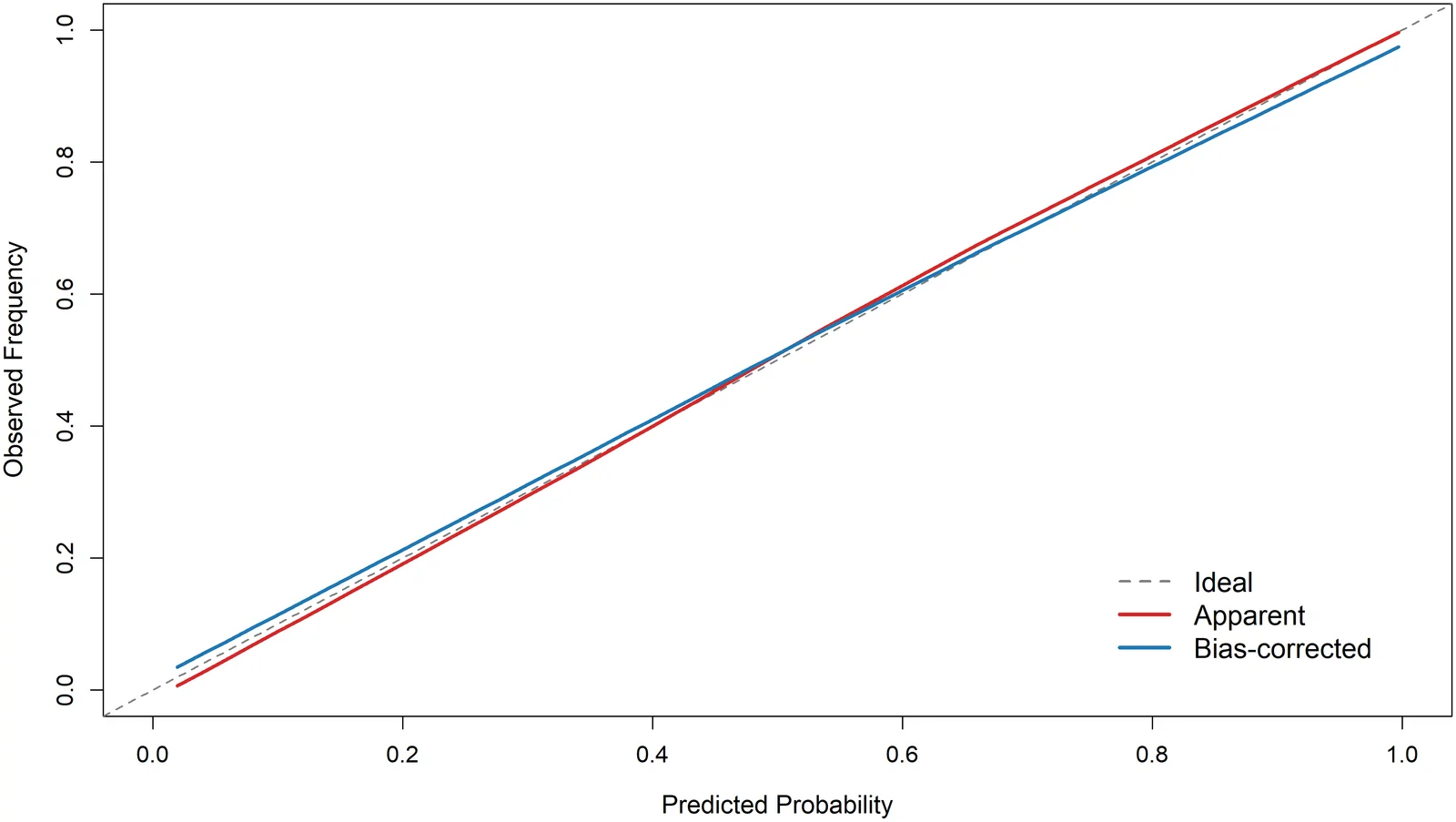
Calibration curve of the logistic regression model for predicting GDM.

Bootstrap resampling (B = 1,000) was used for internal validation and bias correction. The calibration curve showed good agreement between predicted probabilities and observed outcomes (**Figure 3**). The bias-corrected calibration curve remained close to the ideal 45° reference line across the full range of predicted probabilities, suggesting limited overfitting and good generalizability. The Brier score was 0.112 (95% CI: 0.078–0.145), with a standardized Brier score of 0.551, indicating moderate-to-good predictive accuracy. The calibration slope was 1.011 (95% CI: 0.888–1.126), close to the ideal value of 1. Calibration-in-the-large was < 0.0001, and the calibration intercept was −0.006, both close to the ideal value of 0, indicating no substantial systematic bias. The mean absolute error for grouped calibration was 0.084 and the maximum absolute error was 0.200, suggesting balanced predictive accuracy across risk strata. Overall, the model showed good calibration, strong discrimination, and reliable predictive performance, supporting its potential utility for early clinical risk assessment.

The calibration plot evaluates the agreement between the model-predicted probabilities and the observed outcomes. The gray dashed line represents perfect calibration. The red line indicates the apparent (uncorrected) calibration, and the blue line indicates the bias-corrected calibration derived from 1,000 bootstrap resamples. The close alignment of the bias-corrected curve with the ideal line suggests excellent calibration and minimal overfitting.

### Clinical net benefit analysis

**Figure 4:**
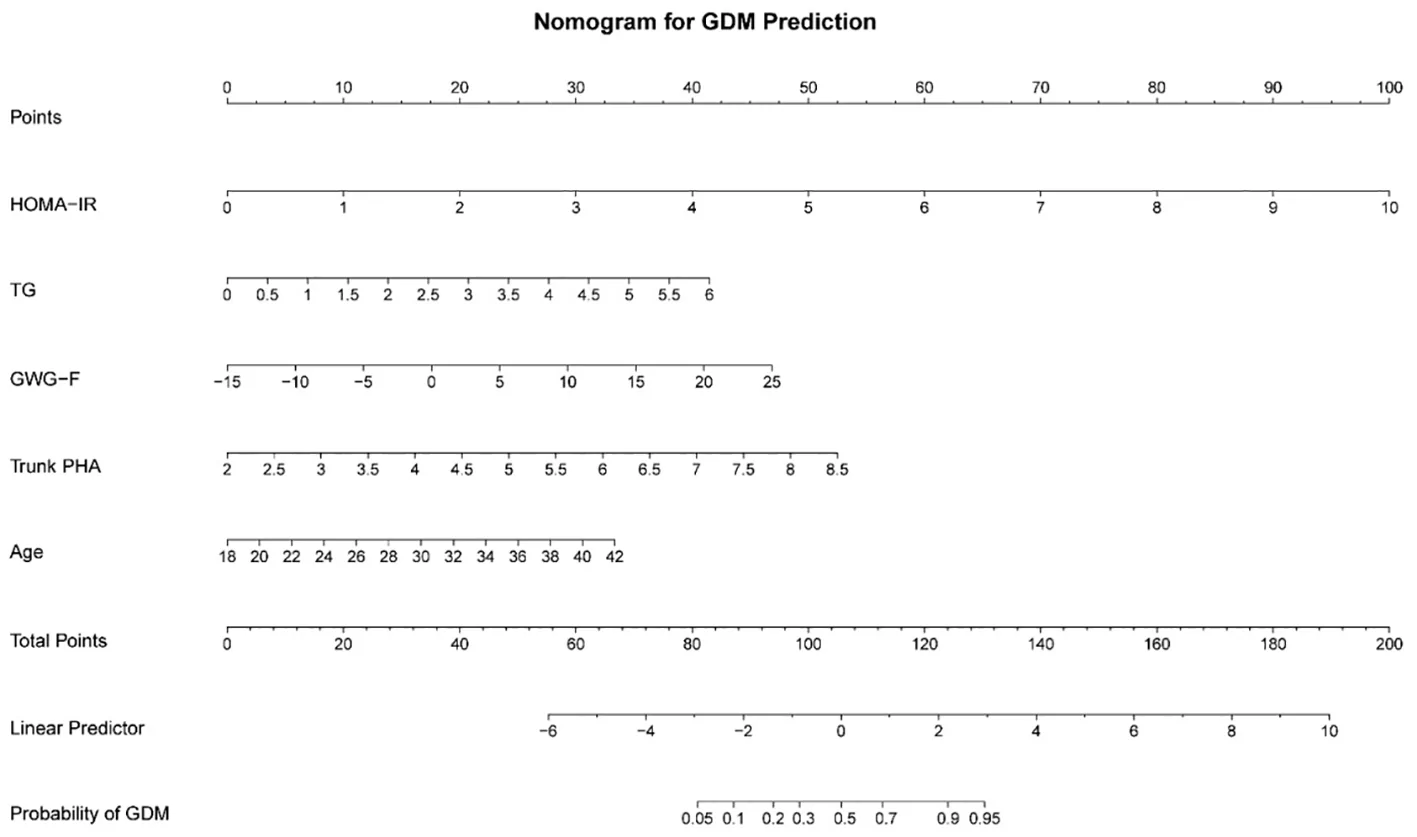
Nomogram for GDM risk prediction.

A nomogram for individualized GDM risk prediction was constructed on the basis of the five independent predictors identified in the multivariable logistic regression analysis, namely HOMA-IR, TG, GWG-F, trunk PhA, and age (**Figure 4**). Each variable was assigned a score according to its regression coefficient, and the total score corresponded to the predicted probability of GDM, thereby providing a practical tool for individualized clinical risk assessment.

**Figure 5:**
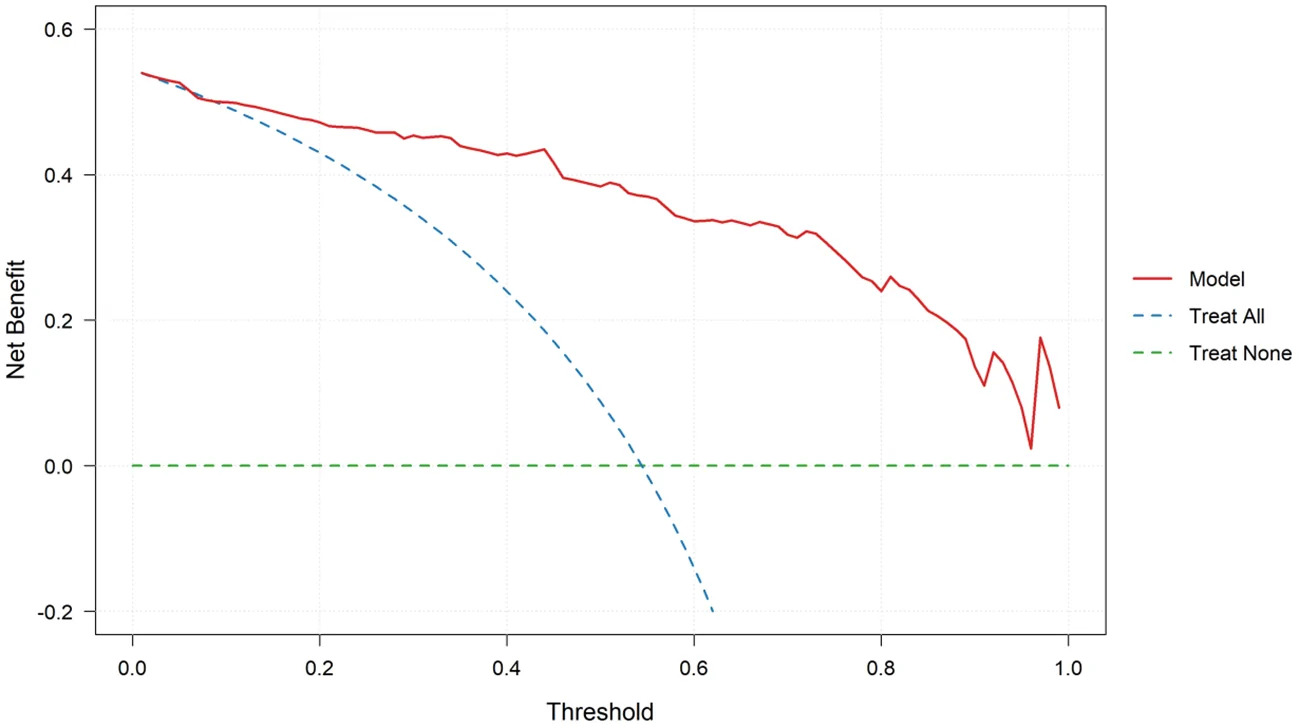
Decision curve analysis of the GDM risk prediction nomogram.

Decision curve analysis was used to evaluate the clinical net benefit and applicability of the nomogram (**Figure 5**). Across a broad threshold probability range of approximately 10%–90%, the nomogram consistently provided greater net benefit than the “treat all” strategy and substantially outperformed the “treat none” strategy. Although some fluctuation in net benefit was observed when the threshold probability exceeded 90%, the nomogram still maintained a positive net benefit. These findings suggest that the model may be clinically useful for guiding GDM screening and early intervention across a wide range of decision thresholds.

The nomogram was developed using five independent predictors: HOMA-IR, TG, GWG-F, trunk PhA, and age. To use the nomogram, locate the value of each variable on its corresponding axis, draw a vertical line upward to the “Points” axis to determine the assigned score, sum all individual scores to obtain the “Total Points,” and then project downward to the “Probability of GDM” axis to estimate the predicted risk of GDM.

## Discussion

GDM is highly prevalent and is closely associated with adverse maternal and neonatal outcomes (24). In current clinical practice, GDM is usually diagnosed in mid-pregnancy (24–28 gestational weeks) by OGTT (23) Therefore, identifying women at high risk during early pregnancy is of considerable clinical importance for preventing pregnancy-related and postpartum complications. In this study, women who subsequently developed GDM already showed marked abnormalities in glucose and lipid metabolism, worsened insulin resistance, and altered body-composition profiles during early pregnancy. After variable reduction based on the EPV principle and multivariable regression analysis, HOMA-IR, TG, GWG-F, 50-kHz trunk PhA, and age were identified as independent risk factors for GDM. The combined model based on these variables showed excellent discrimination (AUC = 0.921, 95% CI: 0.874–0.968), good calibration (Hosmer–Lemeshow P = 0.287), and clear clinical net benefit across a broad range of threshold probabilities. These findings suggest that the model may serve as an efficient and minimally invasive tool for identifying women at high risk of GDM early in pregnancy.

Insulin resistance is a central pathophysiological feature of GDM, and HOMA-IR is one of the most widely used clinical indicators of insulin resistance. In the present study, HOMA-IR was also the best-performing single predictor of GDM (AUC = 0.811) and remained a significant independent risk factor after adjustment for age and pre-pregnancy BMI, consistent with previous studies. Physiological insulin resistance during pregnancy is a compensatory adaptation; however, in women with limited beta-cell reserve, this adaptation may become insufficient, resulting in hyperglycemia and progression to GDM (25). Because HOMA-IR integrates fasting glucose and insulin levels, it provides a useful estimate of insulin sensitivity and has been reported as an independent predictor of GDM not only in mid-to-late pregnancy (26) but also in early pregnancy (27). The significantly higher HOMA-IR observed in the GDM group in our study further supports the central role of insulin resistance in the development of GDM.

Maternal lipid metabolism increases physiologically during pregnancy under the influence of estrogen, progesterone, placental lactogen, and cortisol. This transition reflects a shift from lipogenesis in early pregnancy to lipolysis in late pregnancy, thereby ensuring sufficient energy supply for fetal growth. In women with GDM, these metabolic changes appear to be exaggerated, contributing to both insulin resistance and dyslipidemia (28). Elevated TG and reduced HDL-C have been described as characteristic lipid abnormalities in GDM and may already be present in early pregnancy, suggesting potential value as early biomarkers (29, 30). In the present study, TG, total cholesterol, and LDL-C were significantly higher in the GDM group, which is consistent with previous reports. These early lipid disturbances may impair pancreatic beta-cell function through lipotoxic mechanisms and reinforce a vicious cycle of lipotoxicity and glucotoxicity, ultimately compromising compensation for physiological pregnancy-related insulin resistance (31). Although the TyG index also showed strong predictive performance in univariable analyses, it was not retained in the final multivariable model because of overlap with other composite metabolic indicators. Of note, HDL-C was unexpectedly higher in the GDM group in our cohort, which contrasts with many previous studies. This discrepancy may reflect heterogeneity in age, BMI, or other baseline characteristics. Moreover, increasing evidence suggests that HDL function, rather than absolute HDL-C concentration, may be more informative for disease risk assessment. After adjustment for age, pre-pregnancy BMI, TG, and related confounders, HDL-C was no longer statistically significant, indicating that it was not an independent risk factor for GDM in this cohort.

GWG is an important and potentially modifiable risk factor for GDM. A large Chinese study by Yan et al. (32), which included 9,058 pregnant women, reported that excessive GWG increased GDM risk by approximately 83% (OR = 1.829). Another prospective cohort study (33) showed that excessive GWG increased the risks of GDM, preeclampsia, preterm birth, and macrosomia. In our study, GWG-F was an independent risk factor for GDM, with an AUC of 0.771, sensitivity of 0.618, and specificity of 0.895. This finding is consistent with a birth cohort study from southern China showing that excessive weight gain in early pregnancy is associated with increased GDM risk (34). Excessive early-pregnancy weight gain may reflect energy imbalance and excessive adipose tissue accumulation, thereby aggravating insulin resistance. Importantly, GWG-F showed better predictive performance than a single BMI measurement, suggesting that dynamic monitoring of weight change may be more clinically informative than static anthropometric indicators. Nevertheless, because the sensitivity of GWG-F alone was modest, it should not be used in isolation but rather in combination with other predictors. Excessive GWG is also associated with adverse pregnancy outcomes among women with GDM, including macrosomia, large-for-gestational-age infants, and cesarean delivery (35). Therefore, maintaining appropriate weight gain during early pregnancy may help reduce the occurrence of both GDM and related adverse outcomes.

BIA-based body-composition analysis can provide a comprehensive assessment of nutritional and metabolic status. Among its derived parameters, PhA is considered a marker of cellular integrity, membrane function, and hydration status, and has been proposed as a potential screening indicator for inflammation and oxidative stress (22).

In the present study, 50-kHz PhA values were significantly higher in the GDM group than in the NGT group. This finding differs from observations in type 2 diabetes and obesity, in which lower PhA values are often reported. Hu et al. found that, among patients with type 2 diabetes, those with lower PhA and higher BMI had the most severe insulin resistance, and that PhA was negatively correlated with HOMA-IR (36). Gabiatti et al. also showed that PhA was negatively associated with inflammatory markers such as IL-6 and IL-1β in individuals with metabolic syndrome (37). By contrast, a study by Fu et al. involving 1,729 overweight and obese Chinese participants found that BMI was an independent determinant of PhA, and that PhA increased across BMI strata before declining only at very high BMI levels (38). These findings suggest that the relationship between PhA and BMI may be nonlinear and may vary across different metabolic states.

It is also noteworthy that growing evidence indicates that PhA is independently associated with muscle strength, muscle mass, muscle composition, and body cell mass, highlighting its potential as a surrogate marker of muscle quality (39–42). In our cohort, skeletal muscle mass (SMM), fat-free mass (FFM), and Body Cell Mass (BCM) were all significantly higher in the GDM group than in the NGT group. This difference raises a key question: does the higher PhA in the GDM group reflect a genuine improvement in cellular function, or is it primarily driven by differences in body composition? Because PhA is positively correlated with SMM, FFM, and BCM, and because BMI cannot distinguish between fat and muscle tissue, residual confounding by muscle mass may persist even after adjustment for age and pre-pregnancy BMI in the statistical models. In other words, the elevated PhA in the GDM group may partly reflect greater lean mass rather than a direct pathogenic effect of PhA itself. Therefore, the apparent independent contribution of PhA in the prediction model may partly represent underlying differences in lean tissue mass. Future prediction models should directly measure and adjust for muscle mass, or explore composite indicators such as the PhA-to-muscle-mass ratio, to better define the independent value of PhA in early GDM prediction.

Pregnancy is a unique physiological state characterized by expanded blood volume, shifts in extracellular fluid, and profound hormonal changes, all of which may affect the interpretation of PhA. The higher PhA observed in the GDM group in this study may have several explanations. First, women with GDM may represent an intermediate metabolic state that differs from established type 2 diabetes, and the observed increase in PhA may reflect an early compensatory phase rather than chronic cellular injury. Second, pregnancy-related hemodilution and fluid redistribution may alter the electrical properties measured by BIA. A prospective cohort study has shown that twin pregnancy is associated with lower PhA because of altered fluid distribution (43), further suggesting that pregnancy-specific physiological features should be considered when interpreting PhA. Because evidence on PhA in GDM remains very limited, our finding of elevated trunk PhA in early pregnancy provides a potentially novel clue for future mechanistic investigation. Further studies incorporating inflammatory markers, cytokines, and detailed water-compartment measurements are warranted to clarify the biological significance of PhA in GDM.

The elevated trunk PhA in our GDM cohort may indicate cellular changes associated with insulin resistance and inflammation, yet its interpretation remains confounded by pregnancy-related hemodilution and fluid redistribution. Whether PhA directly reflects GDM-associated cellular dysfunction or primarily captures variations in lean mass requires further investigation. Prospective studies incorporating inflammatory biomarkers and body-water compartment analyses are warranted to validate the predictive value of PhA in early GDM risk stratification.

The combined model constructed in this study, which incorporated HOMA-IR, TG, GWG-F, trunk PhA, and age, achieved an AUC of 0.921, sensitivity of 92.6%, and specificity of 80.7%, clearly outperforming any single predictor. Its performance appears superior to that of many early-pregnancy single-indicator models summarized in previous reviews, which generally reported AUC values ranging from 0.70 to 0.85 (44, 45). Compared with previous GDM prediction models, the present model has several practical strengths: first, it focuses on early pregnancy (8–12 weeks), providing a prediction window approximately 12–16 weeks earlier than conventional OGTT-based diagnosis; second, all included predictors are clinically accessible and minimally invasive, and BIA is radiation-free and repeatable; third, the model is relatively parsimonious, with only a small number of predictors and an EPV of 13.6, which reduces the risk of overfitting. Decision curve analysis further supported the model’s clinical utility, showing a clear net benefit over “treat all” and “treat none” strategies across a wide range of threshold probabilities. These findings suggest that the model may help avoid unnecessary intervention in low-risk women while identifying high-risk women for closer management.

Several limitations of this study should be acknowledged. First, the sample size was relatively small (n = 125), and external validation in larger multicenter cohorts is needed. Second, pre-pregnancy weight was self-reported, which may have introduced recall bias. Third, BIA measurements can be influenced by hydration status, physical activity, and other pre-analytical factors. Fourth, the biological mechanism underlying the observed elevation in PhA remains unclear. Fifth, potentially relevant factors such as ethnicity, physical activity, and dietary intake were not included in the model.

In conclusion, HOMA-IR, TG, first-trimester gestational weight gain, 50-kHz trunk phase angle, and age were identified as independent risk factors for GDM. The combined model demonstrated good discrimination (AUC = 0.921), satisfactory calibration (Hosmer–Lemeshow P = 0.287), and meaningful clinical net benefit, indicating that it may be useful for identifying women at high risk of GDM during early pregnancy (8–12 weeks). Future studies should focus on multicenter prospective validation, elucidation of the biological role of PhA in GDM, development of simplified risk scores or calculators, and evaluation of whether risk-stratified interventions based on this model can improve maternal and neonatal outcomes.

## Data Availability

The raw data supporting the conclusions of this article will be made available by the authors, without undue reservation.

## Supplementary material

Supplementary Table 1 Crude (unadjusted) logistic regression analysis of risk factors for gestational diabetes mellitus.
