# Supplementary material for: Early-pregnancy trunk phase angle derived from bioelectrical impedance analysis for the prediction of gestational diabetes mellitus
Source: Front Endocrinol (Lausanne). 2026 Aug 14;17:1870018. doi: 10.3389/fendo.2026.1870018 (PMC13521792; doi:10.3389/fendo.2026.1870018)
Supplement: Supplementary file 1 [file Table1.docx]

**Supplementary Table S1. Crude (unadjusted) logistic regression analysis of risk factors for gestational diabetes mellitus**

| Variable | B | OR | 95% CI,  Lower95 | 95% CI,  Upper95 | *P* |
| --- | --- | --- | --- | --- | --- |
| FPG, mmol/L | 2.628 | 13.845 | 4.675 | 49.936 | <0.001 |
| TyG index | 1.825 | 6.201 | 2.755 | 15.595 | <0.001 |
| ICW of left arm, L | 3.821 | 45.631 | 1.640 | 1754.678 | 0.031 |
| Left arm fat-free mass, kg | 1.772 | 5.881 | 1.188 | 33.878 | 0.037 |
| TBW of left arm, L | 2.287 | 9.846 | 1.255 | 94.022 | 0.037 |
| Right arm fat mass, kg | 1.427 | 4.166 | 1.101 | 18.403 | 0.048 |
| ECW of left arm, L | 5.614 | 274.191 | 1.254 | 98127.1 | 0.050 |
| ICW of right arm, L | 2.949 | 19.084 | 0.820 | 574.365 | 0.076 |
| Right arm fat-free mass, kg | 1.414 | 4.113 | 0.888 | 21.585 | 0.081 |
| TBW of right arm, L | 1.799 | 6.045 | 0.849 | 50.377 | 0.082 |
| Left arm extracellular water ratio, % | -107.774 | 0.000 | 0.000 | 83962.530 | 0.083 |
| ECW of right arm, L | 4.564 | 95.932 | 0.536 | 25792.06 | 0.095 |
| BMC, kg | 1.301 | 3.673 | 0.759 | 19.559 | 0.114 |
| WHR | -3.755 | 0.023 | 0.000 | 1140.571 | 0.492 |
